# Increased *Nicotiana tabacum* fitness through positive regulation of carotenoid, gibberellin and chlorophyll pathways promoted by *Daucus carota* lycopene β-cyclase (*Dclcyb1*) expression

**DOI:** 10.1093/jxb/erw037

**Published:** 2016-02-18

**Authors:** J.C. Moreno, A. Cerda, K. Simpson, I. Lopez-Diaz, E Carrera, M. Handford, C. Stange

**Affiliations:** ^1^Centro de Biología Molecular Vegetal, Facultad de Ciencias, Universidad de Chile, Las Palmeras 3425, Casilla 653 Ñuñoa, Santiago, Chile; ^2^ Current address: Max Planck Institut für Molekulare Pflanzenphysiologie, Potsdam-Golm, Germany; ^3^Instituto de Biología Molecular y Celular de Plantas, C.S.I.C., Universidad Politécnica de Valencia, Ingeniero Fausto Elío s/n, 46022 Valencia, Spain

**Keywords:** Biomass, carotenoids, chlorophyll, gene expression, gibberellins, lycopene β-cyclase, tobacco.

## Abstract

Carotenoids, chlorophylls and gibberellins are derived from the common precursor geranylgeranyl diphosphate (GGPP). One of the enzymes in carotenoid biosynthesis is lycopene β-cyclase (LCYB) that catalyzes the conversion of lycopene into β-carotene. In carrot, *Dclcyb1* is essential for carotenoid synthesis in the whole plant. Here we show that when expressed in tobacco, increments in total carotenoids, β-carotene and chlorophyll levels occur. Furthermore, photosynthetic efficiency is enhanced in transgenic lines. Interestingly, and contrary to previous observations where overexpression of a carotenogenic gene resulted in the inhibition of the synthesis of gibberellins, we found raised levels of active GA4 and the concommitant increases in plant height, leaf size and whole plant biomass, as well as an early flowering phenotype. Moreover, a significant increase in the expression of the key carotenogenic genes, *Ntpsy1, Ntpsy2* and *Ntlcyb*, as well as those involved in the synthesis of chlorophyll (*Ntchl*), gibberellin (*Ntga20ox*, *Ntcps* and *Ntks*) and isoprenoid precursors (*Ntdxs2* and *Ntggpps*) was observed. These results indicate that the expression of *Dclcyb1* induces a positive feedback affecting the expression of isoprenoid gene precursors and genes involved in carotenoid, gibberellin and chlorophyll pathways leading to an enhancement in fitness measured as biomass, photosynthetic efficiency and carotenoid/chlorophyll composition.

## Highlight

The expression of *Dclcyb1* in tobacco induces a positive feedback on the isoprenoid, carotenoid, gibberellin and chlorophyll pathways leading to an enhancement in fitness, biomass, photosynthetic efficiency and carotenoid/chlorophyll composition.

## Introduction

**Fig. 1.**
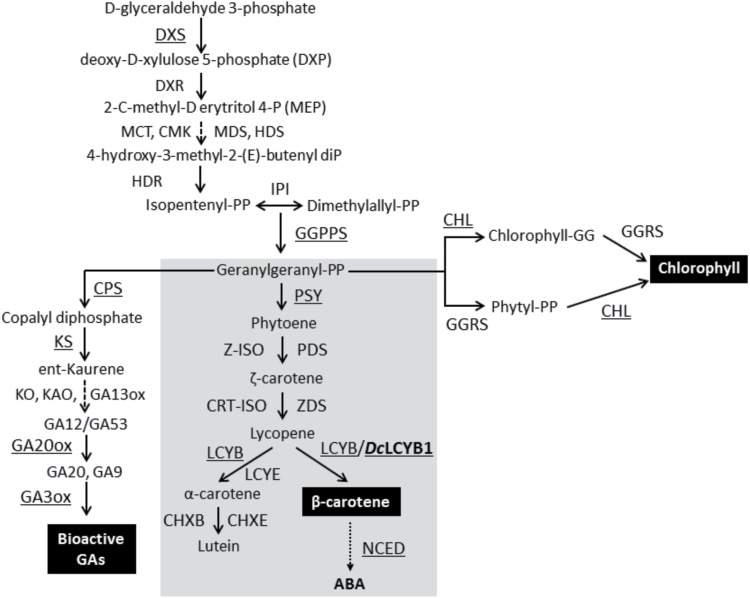
Carotenoid and directly-related biosynthesis pathways. Enzymatic conversions are shown by arrows with the enzymes involved in each reaction. The carotenoid pathway is in the center of the figure enclosed in grey, flanked by the chlorophyll and gibberellin (GA) pathways. Genes analyzed in this work are underlined. The non-mevalonate pathway (MEP) takes place in plastids to produce geranylgeranyl pyrophosphate (GGPP) that is required for carotenoid, chlorophyll and gibberellin synthesis, among others. DXS, deoxyxylulose synthase; DXR, 1-deoxyxylulose 5-phosphate reductoisomerase; CMS, 2C-methyl-D-erythritol 4-phosphate cytidyltransferase, CMK, 4-(cytidine 5-diphospho)-2-C-methyl-D-erythritol kinase, MCS, 2C-methyl-D-erythritol 2,4-cyclodiphosphate synthase, HDS, 4-hydroxy-3-methylbut-2-enyl diphosphate synthase; HDR, 4-hydroxy-3-methylbut-2-enyl diphosphate reductase; IPI, isopentenyl diphosphate isomerase; GGPPS, geranylgeranyl pyrophosphate synthase; PSY, phytoene synthase; PDS, phytoene desaturase; Z-ISO, z-carotene isomerase; ZDS, Z-carotene desaturase; CRT-ISO, carotenoid isomerase; LCYB, lycopene β-cyclase; LCYE, lycopene ε-cyclase; CHXB, β-carotene hydroxylase; CHXE, ε-carotene hydroxylase; GGRS, geranylgeranyl reductase; CHL, chlorophyll synthase; CPS, copalyl diphosphate synthase; KS, ent-kaurene synthase; KO, *ent*-kaurene oxidase; KAO, *en*t-kaurenoic acid oxidase, GA20ox, GA 20-oxidase, GA3ox, GA 3-oxidase; NCED, 9-*cis*-epoxycarotenoid dioxygenase. The LCYB1 enzyme from *Daucus carota* which was overexpressed in this work is boldfaced in the respective reaction of the pathway. The enzymes for which the expression of their corresponding coding sequences was quantitatively analyzed by qRT-PCR are underlined. Dashed lines represent multiple reaction steps.

Carotenoids are the most widespread group of pigments found in nature. More than 750 types of carotenoids are present in bacteria, yeast, fungi, plants and animals (Britton *et al.*, 2004; Takaichi, 2011). In general, carotenoids have high antioxidant activities due to their highly unsaturated backbones (Stahl and Sies, 2003). One of the most widely known carotenoids is β-carotene, which serves, along with α-carotene, as a dietary precursor of vitamin A, which is required for the maintenance of normal vision, cell growth and for healthy immunity, among other physiological processes (Olson, 1996). In plants, these compounds are synthesized in plastids where they play essential roles in light absorption during photosynthesis, photo-protection via energy dissipation, and detoxification of reactive oxygen species (ROS). Carotenoids act as precursors of important apocarotenoids such as the growth regulator abscisic acid (ABA, Fig.1) and strigolactones (Demmig-Adams *et al.*, 1996; Nelson *et al.*, 2003; Van Norman and Sieburth, 2007; Xie *et al.*, 2010), and of volatile flavor/aroma terpenes (Mendes-Pinto, 2009). Moreover, carotenoids synthesized and stored in chromoplasts provide yellow, orange and red colors to fruits and flowers for animal-mediated pollination and seed dispersal. 

The synthesis of carotenoids occurs via the non-mevalonate pathway (MEP; Fig. 1). DXS is the first enzyme in the MEP pathway, and catalyzes the condensation of hydroxyethyl thiamine with glyceraldehyde 3-phosphate to produce 1-deoxy-D-xylulose-5-phosphate (DXP). The second step of the MEP pathway is catalyzed by the enzyme deoxyxylulose 5-phosphate reductoisomerase (DXR), which synthesizes MEP by an intramolecular rearrangement and reduction of DXP. The sequential action of 4-diphosphocytidyl-methylerythritol synthase (CMS), 4-diphosphocytidyl-methylerythritol kinase (CMK) and methylerythritol 2,4-cyclodiphosphate synthase (MCS) allows the conversion of MEP into methylerythritol 2,4-cyclodiphosphate (ME-cPP), which is then transformed to hydroxymethylbutenyl diphosphate (HMBPP) by the hydroxymethylbutenyl diphosphate synthase (HDS) enzyme. Finally, by the action of the enzyme hydroxymethylbutenyl diphosphate reductase (HDR), HMBPP is converted into a mixture of isopentenyl diphosphate (IPP) and dimethylallyl-pyrophosphate (DMAPP) (Fig. 1) (Rodríguez-Concepción and Boronat, 2002; Rodríguez-Concepción, 2010). In plants, DXS, and to a lesser extent DXR and HDR, are rate-limiting steps in isoprenoid synthesis (Estevez *et al.*, 2001; Enfissi *et al.*, 2005; Carretero-Paulet *et al.*, 2006; Morris *et al.*, 2006) and, excluding *Arabidopsis thaliana*, in most species DXS is encoded by a small gene family with members of at least two classes. The first is involved in the biosynthesis of housekeeping isoprenoids, and the second appears to participate in the production of secondary metabolites (Walter *et al.*, 2002). The condensation of three molecules of IPP with one molecule of DMAPP generates the C_20_ molecule geranylgeranyl diphosphate (GGPP), the ubiquitous isoprenoid precursor of carotenoids and several other metabolites, including chlorophylls and gibberellins (Bouvier *et al.*, 2005; Pulido *et al.*, 2012) (Fig. 1). Subsequently, the condensation of two molecules of GGPP yields phytoene (C_40_), which is the first committed step in the carotenoid pathway. Phytoene, a symmetrical colorless linear carotene with only three conjugated double bonds, is then modified to form the reddish lycopene through sequential desaturations and isomerizations carried out by phytoene desaturase (PDS), ζ-carotene desaturase (ZDS), carotenoid isomerase (CRTISO) and ζ-carotene isomerase (ζ-ISO) (Isaacson *et al.*, 2002; Li *et al.*, 2008). The disruption of the carotenoid route at *PDS* impairs chlorophyll, carotenoid, and gibberellin biosynthesis in Arabidopsis and *Nicotiana tabacum* (tobacco) resulting in albino and dwarf phenotypes (Qin *et al.*, 2007). Moreover, two reports related to plastoquinone defective Arabidopsis mutants (*pds1* and *pds2*) that affect phytoene desaturation, also have an albino phenotype (Norris *et al.*, 1995), indicating a fine crosstalk of the carotenoid pathway with other isoprenoid-derived molecules, and the vital requirements of carotenoids for the plant. Lycopene cyclization is accomplished by lycopene β-cyclase (LCYB), which introduces a β-ring at both ends of lycopene producing β-carotene, whereas α-carotene is created by LCYB and lycopene ε-cyclase (LCYE) through the addition of a β-ring followed by the formation of an ε-ring in the other end of the carotene (Beyer *et al.*, 1991; Hornero-Mendez and Britton, 2002; Bang *et al.*, 2007; Misawa, 2011) (Fig.1). Interestingly, carotenoids with β-rings are ubiquitous in nature, whilst carotenoids with ε-rings appear to be restricted to cyanobacteria, algae and land plants (Timmis *et al.*, 2004; Kim and DellaPenna, 2006). Two subsequent hydroxylation reactions on β-carotene, catalyzed by carotenoid β-hydroxylase give rise to β-cryptoxanthin and zeaxanthin, which can be epoxidized by zeaxanthin epoxidase to produce violaxanthin that is converted to neoxanthin by neoxanthin synthase (Cazzonelli and Pogson, 2010; Cazzonelli, 2011; Moise *et al.*, 2014). ABA is produced in the cytoplasm as the final product of this branch, and 9-*cis*-epoxycarotenoid dioxygenases (NCEDs) are involved in this process (Fig. 1).

Carrot (*Daucus carota* L.) is a biennial plant that belongs to the Umbelliferae or Apiaceae family. Depending on the variety of carrot, the storage root has a great diversity of colors, ranging from red, orange or yellow, and even varieties with purple or white roots have been reported (Surles *et al.*, 2004; Arscott and Tanumihardjo, 2010). The diversity of colors between varieties is due to the ability of carrot to synthesize and accumulate large amounts of different types of carotenoids in the root (Surles *et al.*, 2004; Montilla *et al.*, 2011). To date, several carotenogenic genes have been identified in carrot and recently, Moreno *et al.* (2013) showed that the constitutive expression of the carrot lycopene β-cyclase 1 (*Dclcyb1*) gene in carrot produced a significant increase in total carotenoids, β-carotene and chlorophyll in the leaves of transgenic lines. In addition, the level of chlorophyll and carotenoids was impaired when the gene was silenced. Interestingly transgenic carrots with reduced levels of *lcyb1* have a thin colorless storage root, suggesting a relationship between carotenoid accumulation, development and hormone biosynthesis (Moreno *et al.*, 2013).

To further dissect this relationship, here we show that the constitutive expression of *Dclcyb1* in tobacco produces not only an increment in carotenoid, chlorophyll and gibberellin levels, but also benefits the performance of the entire plant, as measured by biomass, plant height, leaf surface, flowering time and photosynthetic efficiency. The phenotypic enhancement is reflected at the molecular level, as transgenic plants have raised expression of isoprenoid precursor, and carotenoid-, chlorophyll- and gibberellin-specific biosynthesis genes. These results permit us to conclude that the improvement in *Dclcyb1* transgenic tobacco fitness is correlated with a positive feedback on carotenoid, chlorophyll and gibberellin synthesis.

## Materials and methods

### 

#### Vector construction

For *Dclcyb1* expression, the complete *Dclcyb1* coding sequence (DQ192190) was cloned into pCR®8/GW/TOPO (Invitrogen) using *DcLcyb*F and *DcLcyb*R (Supplementary Table S1 available at *JXB* online) and following the manufacturer’s instructions. Positive clones obtained by enzymatic digestion were sequenced by Macrogen Corp. (USA). Subsequently, pCR8/*lcyb1* was recombined into pGWB2® (Curtis and Grossniklaus, 2003) to produce the pGWB2-*Dclcyb1* expression vector (Moreno *et al.*, 2013). Positive clones were analyzed through PCR and enzymatic digestion, and transformed into *Agrobacterium tumefaciens* (GV3101 strain).

#### Propagation and transformation of plant material

All tobacco plants (*Nicotiana tabacum* cultivar Xanthi NN) were cultivated in a 16h photoperiod, illuminated with white fluorescent light (150 µmol m^−2^ s^−1^ at 22–25°C) in growth chambers and in a glasshouse located at the Faculty of Science at the University of Chile. Tobacco were cultivated *in vitro* for 6 weeks in solidified Murashige and Skoog (MS; 4.4g l^−1^ MS salts, 20g l^−1^ sucrose and 0.7% agar) medium and transformed according to a previous report (Horsch *et al.*, 1985). Briefly, leaf explants were co-cultivated with *Agrobacterium* carrying pGWB2-*Dclcyb1* or pGWB2 empty vector (e.v.), and placed on solidified MS media containing 1mg l^−1^ BAP, 0.5mg l^−1^ IBA, 25mg l^−1^ kanamycin and 100mg l^−1^ carbenicilin. After 4 weeks, the explants were placed on solidified MS medium supplemented with 50mg l^−1^ kanamycin and 100mg l^−1^ carbenicilin in the absence of hormones to induce the development of transgenic plantlets. When seedlings with proper root development reached 7cm in height, they were transferred to plastic pots (20×10cm) containing a mix of soil and vermiculite (2:1). Fifteen transgenic T_0_ lines were obtained, of which three (L14, L15 and L16) were selected. Plants of the T_1_ and T_2_ of e.v, L14, L15 and L16 were characterized at the molecular and phenotypic level.

#### RNA extraction and quantitative RT-PCR

Total RNA was extracted from frozen powder of tobacco leaves of 8-week-old T_1_ plants using RNAsolv (Omega Biotec, USA). Genomic DNA traces were eliminated by a 20min DNase I treatment. For cDNA synthesis, ~2 μg total DNA-free RNA was mixed with 1mM oligo dT primer and Impron II reverse transcriptase (Promega). Real time RT-PCR experiments were performed with the LightCycler system (Stratagene), using SYBR Green double strand DNA binding dye, as described (Fuentes *et al.*, 2012). Specific primers targeting the codifing region of *DcLcyb1* (*DcLb1* F and *DcLb1* R), as well as for endogenous tobacco genes: *Ntpsy1, Ntpsy2, Ntlcyb, Ntggpps1, Ntdxs1, Ntdxs2, Ntchl, Ntcps, Ntks, Ntga20ox1, Ntga3ox1* and *Ntnced* were designed (for GenBank accession numbers, see Supplementary Table S1). *Actin, ubiquitin* and *18S* (Supplementary Table S1) were initially evaluated as potential normalizers, of which *actin* was selected for further experimental and bioinformatic experiments after analysis by NormFinder (Andersen *et al.*, 2004). Final data was obtained introducing fluorescence results in the equation: Ecarot ΔCP gene (Ct ubiq−Ct gene)/E ubiq ΔCP ref (Ct ubiq−Ct gene) (Pfaffl, 2001). Each qRT-PCR reaction was performed with three biological and two technical replicates. In all cases, the reaction specificities were tested with melting gradient dissociation curves and electrophoresis gels.

#### Pigment extraction and high performance liquid chromatography (HPLC)

Pigments (chlorophyll and carotenoids) were extracted from 100mg of transgenic 8-week-old T_1_ tobacco with 1ml hexane/acetone/ethanol (2:1:1 v/v) as described (Fuentes *et al.*, 2012). Two successive extractions were performed to remove carotenoids until the tissue was blanched. The extract was dried with N_2_ and resuspended in 1ml acetone. Total carotenoids were measured by spectrophotometry at 474nm while chlorophyll *a* and *b* quantification was performed at 474nm (absorption of carotenoids plus chlorophyll), 645nm (chlorophyll *a*) and 662nm (chlorophyll *b*). Pigments were separated in a Shimadzu HPLC (LC-10AT) equipped with a diode array detector using a RP-18 LiChroCART 125-4 reverse phase column (Merck, Germany), and a mix of acetonitrile:methanol:isopropanol (85:10:5 v/v) as a mobile phase. Separation was carried out with a 1.5 m min^−1^ flow rate at room temperature under isocratic conditions. Results were analyzed with the LCsolutions software package (Shimadzu, USA). The elution spectra of each maximum were obtained using a diode array detector. The carotenoids were identified according to the absorption spectra, retention time and comparison with specific standards, which were corroborated by comparison with the *Carotenoids Handbook* (Britton *et al.*, 2004). All operations were carried out with three biological replicates, on ice and in the dark to avoid photodegradation, isomerization and/or structural changes of pigments.

#### Plant fitness analysis

Tobacco T_0_, T_1_ and T_2_ plants were subjected in a randomized design (*n*=7) to morphological measurements (number of total leaves produced, leaf length and width, as well as nodal interspace and stem diameter) at 3 months when plants reached maturity. Plant height was measured from the flowering apex to the base after the flowering apex became visible. The area of detached leaves from node 4 of every plant was estimated by multiplying the length and the width of each leaf. Stem diameter was measured below node 5 (node 0 was determined as the first leaf with length >7cm). Internode distance was measured between nodes 3 and 4, nodes 4 and 5 and nodes 5 and 6. Biomass (fresh and dry weight) and seed yield (dry weight in g) were also determined. Fresh and dry weight were recorded at maturity (at the onset of flower bud formation, at 3 months in transgenic L14, L15 and L16 and at 4 months in e.v.). The leaves and stems (>5cm above soil level) were harvested, weighed immediately to determine fresh weight and dried at 70°C for 48h (or until no further decrease in weight could be detected) to determine dry weight. Seeds were harvested and weighed 1 month after flowering when seed drying was achieved.

#### Chlorophyll fluorescence measurements

Chlorophyll fluorescence was measured using an Fms1 fluorometer (Hansatech, UK). For the determination of maximum quantum yield of photosystem (PS) II, plants were preincubated for 30min in the dark. Minimum fluorescence yield (F_0_) was measured by applying a weak pulsed red light (<1 μmol quanta m^−2^ s^−1^). Maximum fluorescence yield (F_m_) was determined during a 0.8s pulse of white light of intensity 3.500 μmol quanta m^−2^ s^−1^. Maximum quantum yield of PS II was calculated as (F_m_−F_0_)/F_m_=F_v_/F_m_, where F_v_ corresponds to F_m_−F_0,_ the maximum variable of chlorophyll fluorescence yield in the dark-adapted state. All measurements were performed in three different leaves from the distal nodes with three technical replicates. Ten plants from the T_1_ generation were used per line.

#### Gibberellin synthesis inhibition assay

In order to perform the gibberellin inhibition assay, the compound AMO1618 was used. This chemical impairs the early steps of the gibberellin biosynthesis pathway, by specifically blocking the copalyl diphosphate synthase (CPS) and ent-kaurene synthase (KS) enzymes (Kawaide *et al.*, 1997). The effect of the inhibitor was analyzed through quantification of leaf surface area and plant biomass, in terms of fresh weight, in 1-month-old T_1_ tobacco plants grown in MS media in the presence of kanamycin (100mg l^−1^) and AMO1618 (100 µM) (Supplementary Fig. S3). These assays were undertaken in three biological replicas, each with 16 tobacco plants per line, and the results analyzed by ImageJ (NIH).

#### Hormone quantification

Aliquots of 200mg of fresh leaf tissue of 1-month-old T_1_ tobacco plants were ground and lyophilized. The material was resuspended in 80% methanol/1% acetic acid, containing [17,17-^2^H]GAs and [^2^H_6_]ABA as internal standards, mixed by shaking for 1h at 4ºC and kept at −20ºC overnight. The extract was passed through an Oasis HLB (reverse phase) cartridge as described (Seo *et al.*, 2011). The dried eluate was dissolved in 5% acetonitrile/1% acetic acid, and the GAs and ABA were separated by UHPLC (Accucore RP-MS column 2.6 µm, 50×2.1mm, Thermo Scientific, UK) with a 5–50% acetonitrile gradient containing 0.05% acetic acid, at 400 µl min^−1^ over 14min. The concentrations in the extracts were analyzed with a Q-Exactive mass spectrometer (Orbitrap detector; ThermoFisher Scientific, MA, USA) by targeted SIM using embedded calibration curves and the Xcalibur 2.2 SP1 build 48 and TraceFinder programs.

#### Statistical analysis

To test for significant differences in gene expression, physiological parameters and hormone levels, ANOVA (with Tukey’s post-test) or one- and two-tailed Student’s *t*-tests were carried out using the General Linear Models option in the statistical software package Graphpad Prism.

## Results

### Transgenic tobacco lines that express *Dclcyb*1 show an increment in total carotenoids, β-carotene and lutein in correlation with enhanced expression of endogenous carotenogenic genes

**Fig. 2.**
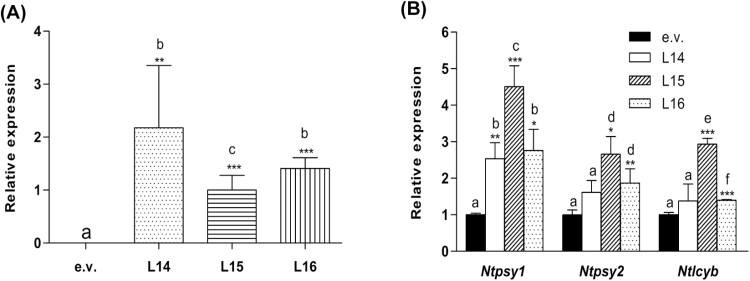
Relative expression levels of *Dclcyb1* and key endogenous carotenoid genes in transgenic tobacco lines. (A) Relative expression levels of *Dclcyb1* in leaves of empty vector (e.v.) and *Dclcyb1* transgenic (L14, L15 and L16) 8-week-old T_1_ tobacco plants. (B) Relative expression of endogenous *Ntpsy1*, *Ntpsy2* and *Ntlcyb*. *Actin* was used as normalizer in qRT-PCR measurements. Columns and bars represent the mean and SE (*n*=6). Asterisks indicate significant differences between *Dclcyb1* transgenic lines and the e.v. transformants. Non-paired two-tailed Student’s *t*-tests were performed for all transgenic lines: *, *P*<0.05; **, *P*<0.01; *** *P*<0.001.

**Fig. 3.**
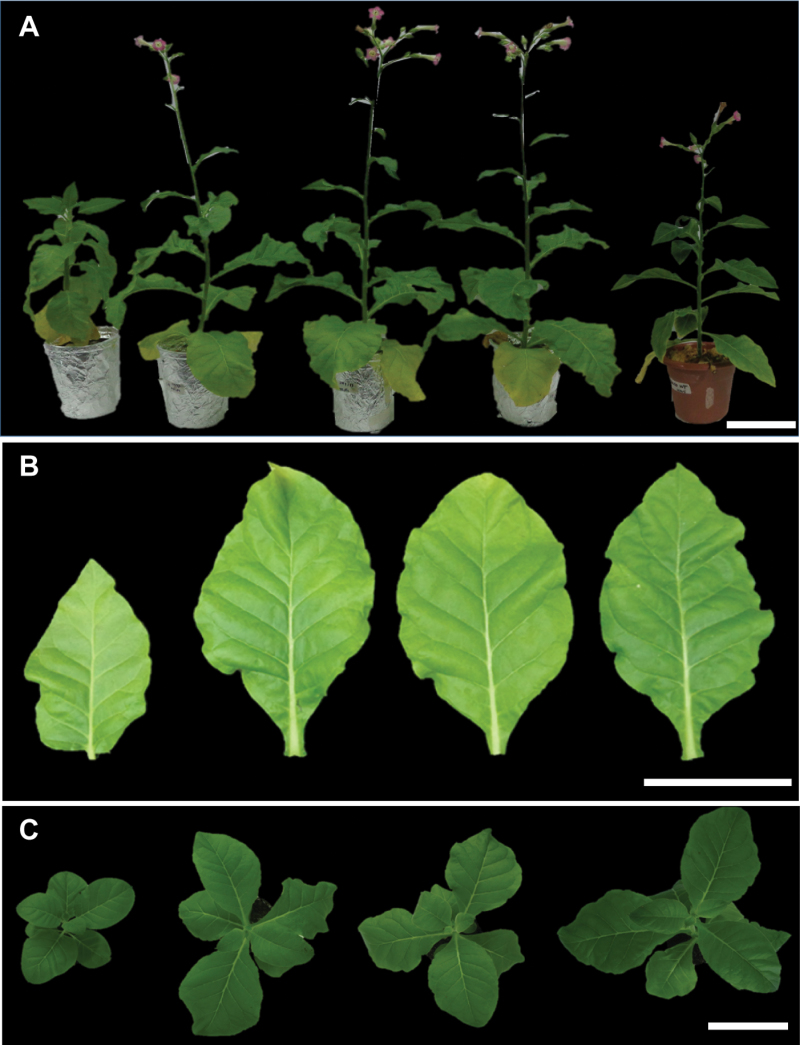
Phenotype of transgenic *Dclcyb1* tobacco lines. (A) Left: representative 12-week-old T_1_ empty vector (e.v.), centre: representative 12-week-old T_1_ *Dclcyb1* transgenic (L14, L15 and L16) tobacco lines, right: representative 16-week-old T_1_ e.v. line. (B) Detached leaves correspond to the 4th node (from the base of the plant) of plants shown in panel A. (C) Representative 8-week-old T_1_ e.v. and *Dclcyb1* transgenic tobacco lines are shown. Scale bar, 10cm.

To alter carotenoid levels in tobacco plants, the coding sequence of the *lcyb1* gene from carrot (*Dclcyb1*) was cloned under the direction of the 35S promoter. Plants were transformed and 15 T_0_ lines were obtained. Three of these lines (L14, L15 and L16) showed significant increases in β-carotene and chlorophyll levels as well as in plant height (Supplementary Fig. S1, Supplementary Table S2). Seeds from these three lines were collected and T_1_ plants were selected by their kanamycin resistance. Three-month-old T_1_ transgenic lines were subjected to further molecular, phenotypic and physiological characterization. Six biological replicates from pools of each line were subjected to qRT-PCR analysis and *Dclcyb1* gene expression was determined; transcript abundance was greatest in line L14 and L16 (Fig. 2A). Representative phenotypes of transgenic plants are shown in Fig. 3 in which a noticeable qualitative difference in height and flowering time can be observed between *Dclcyb1* lines respect to e.v.; flower bud formation in *Dclcyb1* lines was achieved in 3 months, one month earlier than e.v.

Additionally, we measured the pigment content (Table 1) to evaluate whether the expression of *Dclcyb1* induces accumulation of carotenoids and especially of β-carotene in transgenic tobaccos. Transgenic T_1_ lines showed an increase of 2- to 2.2-fold in β-carotene levels respect to e.v. (Table 1). Interestingly, we also observed a 1.8- to 2.2-fold increase in lutein levels (Table 1), which is found in the other branch of the carotenoid pathway (Fig. 1), indicating that the expression of the *Dclcyb1* gene up-regulates both branches of the pathway. These findings are also reflected in the significant increase in total carotenoid levels (composed mostly of β-carotene and lutein) that we observed in *Dclcyb1* transgenic lines (Table 1). To further explore these results, the expression of the key endogenous carotenogenic genes, *Ntpsy* and *Ntlcyb*, was analyzed (Fig. 2B). We determined that the transcript levels of both copies of tobacco *psy* (*Ntpsy1* and *Ntpsy2*) were raised between 2.5- and 4.5-fold in all lines (*Ntpsy1*), and by 1.8- (L15) and 2.5-fold (L16) for *Ntpsy2*. In addition, the endogenous *Ntlcyb* expression level was increased between 1.4- and 3-fold in L15 and L16 (Fig. 2B). Therefore, these results indicate the operation of a general up-regulatory mechanism in the expression of key endogenous carotenogenic genes in the transgenic lines, suggesting that a molecular positive feedback system is triggered by the expression of *Dclcyb1*.

**Table 1.** Carotenoid and chlorophyll content in T_1_ *Dclcyb1* transgenic lines

Lines	Lutein (μg g^−1^ DW)	β-carotene (μg g^−1^ DW)	Tot. carot. (μg g^−1^ DW)	Chl a (μg g DW)	Chl b (μg g^−1^ DW)	Chl:Car ratio
e.v.	68.29±8^a^	342±10^a^	424±32^a^	1846±324^a^	827±324^a^	6.3±1.46^a^
L14	**140±18** ^**b**^	**673±26** ^**b**^	**835±32** ^**b**^	**4013±461** ^**b**^	973±471^a^	5.9±0.47 ^a^
L15	**126±4** ^**b**^	**741±4** ^**b**^	**884±112** ^**b**^	**4105±458** ^**b**^	798±209^a^	5.5±0.01^a^
L16	**151±17** ^**b**^	**747±20** ^**b**^	**923±77** ^**b**^	**3751±489** ^**b**^	1286±225^a^	5.5±0.74^a^

Carotenoid and chlorophyll content are presented as μg g^−1^ dry weight for all the transgenic lines (e.v., L14, L15 and L16). Measurements were made from seven plants per line, using a pool of four representative leaves per plant. ^a^, ^b^: Non-paired two-tailed Student’s *t*-test (*P* < 0.05) were performed for all transgenic lines. Differences are represented by letters and significant values are boldfaced. The mean data are presented ± SD (*n*=7). e.v., empty vector; Chl a, chlorophyll a; Chl b, chlorophyll b; Chl, Chl a + Chl b; Car, total carotenoids (β-carotene+lutein+others).

### Tobacco plants expressing *Dclcyb1* present an increase in chlorophyll and photosynthetic parameters

We observed that all *Dclcyb1* tobacco lines showed a 2–2.2-fold increase in chlorophyll *a* (Table 1), consistent with coordinate regulation of carotenoid and chlorophyll synthesis (Toledo-Ortiz *et al.*, 2010; Cheminant *et al.*, 2011; Stange and Flores, 2011). To gain additional insights into the consequences of the increase in chlorophyll levels, we measured the F_v_/F_m_ ratio, which reflects photosynthetic efficiency in terms of chlorophyll fluorescence. As shown in Table 2, the F_v_/F_m_ ratio is significantly higher in all three lines analyzed compared to e.v., in a direct correlation with total chlorophyll and chlorophyll a/b ratio. The Chl:Car ratio, another photosynthetic and metabolic parameter, is similar between all *Dclcyb1* lines due to the proportional increases in both pigments compared to e.v. controls (Table 1).

**Table 2.** Photosynthesis parameters in T_1_ *Dclcyb1* transgenic lines

Lines	F_v_/F_m_	Total chlorophyll (μg g^−1^ DW)	Chlorophyll a/b
e.v.	0.745±0.1^a^	2673±742^a^	2.5±1.0^a^
L14	**0.861±0.02** ^**b**^	**4986±887** ^**b**^	4.6±1.7^a^
L15	**0.844±0.03** ^**b**^	**4903±617** ^**b**^	**5.3±1.2** ^**b**^
L16	**0.866±0.01** ^**b**^	**5037±412** ^**b**^	3.0±0.9^a^

^a^, ^b^: non-paired one- and two-tailed Student’s t-tests (*P* < 0.05) were performed for all the transgenic lines (e.v., L14, L15 and L16). The differences are represented by letters and significant values are boldfaced. Measurements were made from ten different plants per line, using a pool of three representative leaves per plant for F_v_/F_m_ ratio and three different plants per line for total chlorophyll quantification. The mean data are presented ±SD (*n*=10) and (*n*=3). e.v., empty vector; DW, dry weight.

**Fig. 4.**
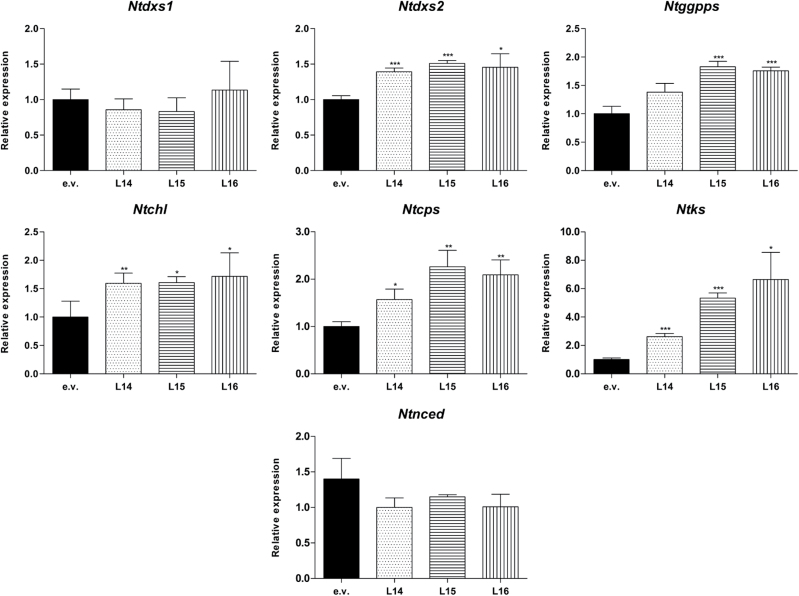
Relative expression of key genes involved in isoprenoid precursors, chlorophyll, gibberellins and ABA synthesis pathways. Relative expression of *Ntdxs1*, *Ntdxs2* and *Ntggpps* genes that codify for key enzymes acting in the synthesis for carotenoid, gibberellin and chlorophyll precursors, as well as *Ntchl* (chlorophyll synthesis), *Ntcps* and *Ntks* (gibberellin synthesis) and *Ntnced* (ABA synthesis) was measured in leaves of 2-month-old T_1_ empty vector (e.v.) and *Dclcyb1* transgenic (L14, L15 and L16) tobacco lines. *Actin* was used as normalizer in qRT-PCR measurements. Columns and bars represent the means and SE (*n*=6). Asterisks indicate significant differences between *Dclcyb1* transgenic lines and e.v. transformants, as determined by non-paired two-tailed Student’s t-tests. *, *P*<0.05; **, *P*<0.01; ***, *P*<0.001.

Interestingly, chlorophyll and photosynthesis efficiency correlates directly with a 1.5–1.7-fold increase in chlorophyll synthase gene (*Ntchl*) expression levels (Fig. 4). This could be due to a feedback mechanism triggered by the expression of *Dclcyb1* on endogenous chlorophyll biosynthesis genes, as found for endogenous carotenogenic genes (Fig. 2B). These results suggest that the transgenic lines are performing or obtaining more products from the photosynthetic process in order to increase their fitness.

### Tobacco plants with higher *Dclcyb1* expression levels show an increase in biomass and fitness

We determined other fitness parameters such as plant height, leaf surface area and biomass (fresh and dry weight) when transgenic plants reached maturity. Maturity is defined when the flowering apex becomes visible, i.e. at 3 months in transgenic plants (Fig. 3). All the T_1_ tobacco transgenic lines (L14, L15 and L16) showed an increase of ~2-fold in plant height, which is also related with the increase in internode space compared to e.v. (Table 3). Leaf length and width were also higher in these lines, with an increase in >2-fold in estimated leaf surface area (Table 3). Interestingly, leaf number was reduced in all the transgenic lines compared to the e.v. plant, whilst stem diameter was unaffected (Table 3). In terms of biomass, the transgenic lines showed an increase of 1.3–1.5-fold in fresh weight of the leaves and 1.6–1.8-fold for the stem, reflected in 1.4– 1.7-fold increases in the whole plant fresh weight (Table 4). However, even though these lines presented no significant changes in the dry weight of the leaves, they presented a significant increment in the dry weight of the stems (1.3-fold compared to e.v.; Table 4). These results suggest that different metabolic pathways related with photosynthesis, plant growth and development measured as biomass are being altered, causing a positive effect in the fitness of *Dclcyb1*-expressing tobacco plants.

**Table 3.** Fitness parameters in T_1_ *Dclcyb1* transgenic lines

Lines	Plant height (cm)	Stem diameter (cm)	Leaf length (cm)	Leaf width (cm)	Leaf surface (cm^2^)	Nodal interspace (cm)	Leaf number
e.v.	28.6^a^	0.79^a^	13.4^a^	7.8^a^	104.8^a^	2.1^a^	22^a^
L14	**55.8** ^**b**^	0.75^a^	**19.2** ^**b**^	**11.2** ^**b**^	**215.3** ^**b**^	**3.0** ^**b**^	**15** ^**b**^
L15	**56.2** ^**b**^	0.76^a^	**19.0** ^**b**^	**11.2** ^**b**^	**213.8** ^**b**^	**3.0** ^**b**^	**14** ^**b**^
L16	**54.0** ^**b**^	0.76^a^	**18.7** ^**b**^	**11.0** ^**b**^	**207.0** ^**b**^	**2.9** ^**b**^	**14** ^**b**^

^a, b^: non-paired two tailed Student’s *t*-tests (*P* < 0.05) were performed for all the transgenic lines (e.v., L14, L15 and L16). The differences are represented by letters and significant values are boldfaced. Three representative leaves from seven different plants were used. The mean data are presented (*n*=7). e.v., empty vector.

**Table 4.** Biomass measurements in T_1_ *Dclcyb1* transgenic lines

Lines	Biomass FW/ leaf (g)	Biomass FW/ stem (g)	Biomass FW/ plant (g)	Biomass DW/ leaf (g)	Biomass DW/ stem (g)	Biomass DW/ plant (g)	Seed production/ plant (g)
e.v.	16.87^a^	11.04^a^	27.51^a^	1.93^a^	1.76^a^	3.69^a^	0.781^a^
L14	**26.04** ^**b**^	**19.49** ^**b**^	**45.54** ^**b**^	2.57^a^	**2.30** ^**b**^	**4.9** ^**b**^	**1.27** ^**b**^
L15	**22.04** ^**b**^	**17.90** ^**b**^	**39.94** ^**b**^	1.87^a^	**2.27** ^**b**^	4.14^a^	**1.41** ^**b**^
L16	**21.91** ^**b**^	**17.66** ^**b**^	**39.58** ^**b**^	1.85^a^	**2.25** ^**b**^	4.11^a^	**1.44** ^**b**^

^a, b^: Non-paired one- and two-tailed Student’s *t*-tests (*P* < 0.05) were performed for all the transgenic lines (e.v., L14, L15 and L16). The differences are represented by letters and significant values are boldfaced. The mean data are presented (*n*=7). FW, fresh weight; DW, dry weight; e.v., empty vector.

### *Dclcyb1* tobacco lines with greater biomass and fitness possess higher gibberellin levels

Although the increase in biomass and fitness can be explained by the up-regulation in key genes in the carotenoid and chlorophyll pathway, we also examined the expression of copalyl phosphate synthase (*Ntcps*), ent-kaurene synthase (*Ntks*), GA20 oxidase (*Ntga20ox*) and GA3 oxidase (*Ntga3ox*), key genes of the gibberellin biosynthesis pathway that share the same GGPP precursor with the carotenoid and chlorophyll pathways (Fig. 1). Gibberellin is a hormone involved in cell and organ enlargement (Bouvier *et al.*, 2005), and could therefore be associated with the greater plant and leaf size as well as the increment in biomass and fitness presented by the transgenic plants (Fig. 3, Table 3). The overexpression of genes involved in GAs biosynthesis induces hypocotyl elongation, early flowering and a rise in bioactive gibberellins (Coles *et al.*, 1999; Daviere and Achard, 2013). For instance, *Dclycb1*-expressing transgenic plants flower a month before e.v. Additionally, at flowering, transgenic and e.v. plants reached 55cm and 41cm in height, respectively (Fig. 3) and transgenic plants produced significantly more seeds than e.v. (Table 4). For relative expression analysis, we found an increase of between 1.4- and 2.3-fold in the expression levels of *Ntcps* and a significant 2.5–6-fold induction of *Ntks* in all *Dclcyb1* transgenic lines compared to e.v. (Fig. 4). Additionally, T1 transgenic plants present a 3–4-fold increment for *Ntga20ox* transcript abundance in L15 and L16 while *Ntga3ox1* is not significantly induced in L14 and L16 (Supplementary Fig. S3), in agreement with the findings of Gallego-Giraldo *et al.* (2008) that showed that GA3oxs are non-limiting enzymes in gibberellin synthesis in tobacco. These results are in a direct correlation with the rise in height, leaf size, early flowering and seed production obtained for *Dclcyb1* transgenic lines (Tables 3, 4).

**Fig. 5.**
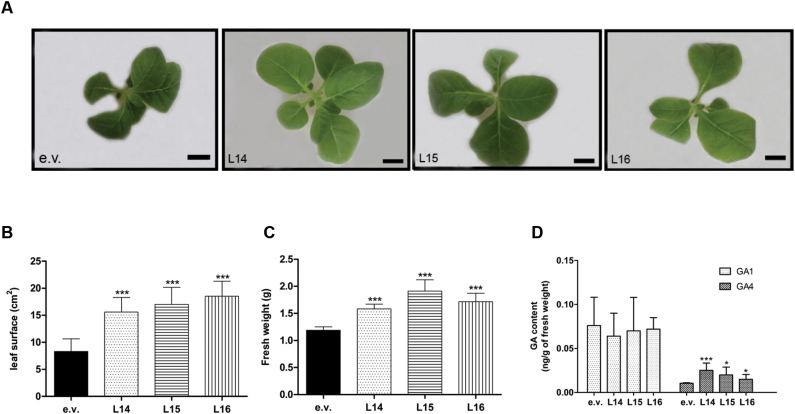
Gibberellin levels in transgenic tobacco plants. (A) Phentotype of 1-month-old empty vector (e.v.) and *Dclcyb1* transgenic (L14, L15 and L16) T_1_ tobacco lines. (B) Fresh weight and (C) leaf surface area for 1-month-old e.v., L14, L15 and L16 transgenic lines. Columns and bars represent the means and SE (*n*=16). Letters indicate significant differences between transgenic lines and the e.v. plants as determined by ANOVA analysis with Tukey’s post-test, *P*<0.0001. Scale bar, 2cm. (D) The bioactive gibberellin (GA1 and GA4ng/g FW) contents in e.v. and *Dclcyb1* transgenic T_1_ tobacco lines are shown. Three biological replicates and two technical replicates from leaves of 1-month-old tobacco plants were used. Asterisks indicate significant differences between *Dclcyb1* transgenic lines and the e.v. transformants, as determined by non-paired two-tailed Student’s *t*-tests, *, *P*<0.05; ***, *P*<0.001. (This figure is available in colour at *JXB* online.)

To confirm directly the involvement of gibberellins in the enhanced performance of the transgenic plants, we quantified the bioactive gibberellins in 1-month-old *Dclcyb1* transgenic lines that showed the typical increase in plant fresh weight and leaf surface compared to the e.v. plants, presenting a 2–2.5-fold increase in leaf area compared with e.v. (Fig. 5A–C). In tobacco plants, gibberellins are found in two bioactive forms, GA1 and GA4 (Hedden and Kamiya, 1997; Hedden and Phillips, 2000). LC-MS analysis showed that GA1 accumulated in all lines and no significant differences between the e.v. and the *Dclcyb1* transgenic plants were found (Fig. 5D). Interestingly, the GA4 content was 2.5-, 2- and 1.6-fold higher in L14, L15 and L16 *Dclcyb1* transgenic lines, respectively, compared to e.v. (Fig. 5D). Moreover, a GA inhibition assay was performed using AMO1618, which blocks CPS and KS enzymes in the initial steps of the GA synthesis pathway (Kawaide *et al.*, 1997). After 1 month of culture *Dclcyb1* transgenic lines suffered a 50–55% reduction in fresh weight, whilst that of the e.v. control line presented a more substantial 67% reduction (Supplementary Fig. S4B). Moreover, the same behavior was observed in tobacco seedlings growing in vertical plates for 3 weeks in the presence of AMO1618 (Supplementary Fig. S4D). All *Dclcyb1* transgenic lines treated with AMO1618 suffered a significant reduction in leaf area, reaching the same size as untreated e.v. plants (Supplementary Fig. S4C), although primary root length remained unaffected (Supplementary Fig. S4E). These results support our proposal and allow us to conclude that the higher biomass, early flowering and greater plant and leaf size reached by the transgenic lines expressing *Dclcyb1* is due to the higher levels of GA4 produced in combination with higher carotenoid and chlorophyll levels. This hypothesis is supported by the fact that in general, *Dclcyb1*-expressing lines have greater expression levels of *Ntdxs2* and *Ntggpps* (Fig. 4), which code for enzymes involved in the synthesis of GGPP, the common precursor of the gibberellin, chlorophyll and carotenoid biosynthetic pathways (Fig. 1).

## Discussion

### Expression of *Dclcyb*1 produces an increment in carotenoid and chlorophyll levels through the up-regulation of endogenous genes

Transgenic tobacco lines display an increment in *Dclcyb1* expression in direct relation with increases in total carotenoids, β-carotene and lutein. Similar results were previously obtained for the T_0_ generation (Supplementary Fig. S1). This suggests that the effect of *Dclcyb1* is stably-inherited by the progeny. Considering that *Dclcyb* gene product is involved in the formation of β-carotene and lutein (Fig. 1), the increase in these carotenoids is expected, and indicates that *Dclcyb1* promotes the synthesis of pigments of both branches. Moreover, this also suggests that *Ntlcye* and *Ntchx* are not limiting steps in lutein biosynthesis. Furthermore, the expression of *Dclcyb1* induces a higher transcript accumulation of key endogenous genes, such as *Ntpsy1*, *Ntpsy2* and *Ntlcyb* (Fig. 2). The same effect was observed in carrot where transcripts of endogenous *Dcpsy1*, *Dcpsy2* and *Dclcyb2* genes were increased when this gene was overexpressed in this organism (Moreno *et al.*, 2013). A similar feedback mechanism was obtained in tobacco *Ntlcye* knockdown plants where transcripts of endogenous *Ntpsy* and *Ntlcyb* genes, among others, where up-regulated (Shi *et al.*, 2014) or in cosuppressed Arabidopsis *psy* plants in which the expression of the *or* gene was reduced, probably due to chloroplast impairment or by retrograde signaling transcriptional regulation of *or* (Zhou *et al.*, 2015). A reduction in *psy* transcript level was observed when *Helianthus annuus Haggpps*, involved in the synthesis of the GGPP isoprenoid precursor, is expressed in tobacco and Arabidopsis (Tata *et al.*, 2015). Moreover, in a T-DNA insertion mutant of *pds3* in Arabidopsis, similar feedback effects on the expression of endogenous genes was observed (Qin *et al.*, 2007). The *pds3* mutant showed a dwarf albino phenotype accompanied by decreased carotenoid, chlorophyll and gibberellin levels. In this case, the disruption of *pds3* causes not only a decrease in *Atpsy*, *Atzds*, and *Atlcyb* expression, but also in genes involved in carotenoid precursor formation, such as *Atggpps* and *Atipi*, as well as in chlorophyll *Atggrs* and in gibberellin *Atcps* precursor genes (Qin *et al.*, 2007). Similar results were obtained by Busch *et al.* (2002) after overexpressing *Ntpsy* or *Ntpds* in tobacco. Taken together, this body of evidence suggests the presence of a negative feedback route given by high transcript abundance, regulating not only the carotenoid pathway but also the chlorophyll and gibberellin pathways. In our work, we observed a positive feedback mechanism when the *Dclcyb1* gene is expressed twofold in tobacco, leading to an increase in transcript levels in carotenoid (*Ntlcyb*, *Ntpsy1* and *Ntpsy2*), carotenoid-precursors (*Ntggpps* and *Ntdxs2*), chlorophyll (*Ntchl*) and gibberellin (*Ntcps* and *Ntks*) genes.

Additionally, tobacco lines possess a significant increase in chlorophyll *a* content (Table 1). Such a concomitant increment in carotenoid and chlorophyll content has also been reported in leaves of carrot that overexpress *Dclcyb1* (Moreno *et al.*, 2013) and in seeds of Arabidopsis that express *psy* (Lindgren *et al.*, 2003), which suggests a direct crosstalk between the carotenoid and chlorophyll pathways (revised in Stange and Flores, 2011). Furthermore, *pds* gene silencing in tobacco plants reduces both chlorophyll and carotenoid content, but also causes albinism, and PS II malfunction (Wang *et al.*, 2009). This can be explained by the fact that both pigments share GGPP as the common precursor and because chlorophyll and carotenoids (specifically, β-carotene and lutein) are required in several pigment-protein complexes in the reaction centers of PSI and II (Bassi *et al.*, 1993). Crosstalk between both pathways through DELLA proteins has also been reported to promote cotyledon greening in dark and de-etiolated seedlings (Cheminant *et al.*, 2011). It is important to note that in all *Dclcyb1* transgenic lines, the Chl:Car ratio is similar to e.v. plants due to the fact that both pigments increase proportionally in *Dclcyb1* expressing plants. The significant increase in chlorophyll *a* agrees with the observed up-regulation of the chlorophyll synthase gene (*Ntchl*) involved in chlorophyll synthesis (Fig. 4) and the greater photosynthetic efficiency, as measured by F_v_/F_m_ (Table 2), which could be responsible in part for the significantly higher biomass and fitness parameters observed in these transgenic plants (Fig. 3, Table 3). Biomass and fitness parameters, measured as plant height, stem diameter, leaf length, width and estimated surface area, as well as internodal space, leaf number and early flowering were significantly higher in T_0_, T_1_ and T_2_ generations of *Dclcyb1* transgenic plants (Table 3, Supplementary Tables S2–S4). These results reaffirm that the effect produced by *Dclcyb1* is stably inherited through to the next generation.

### *Dclcyb1* produces an up-regulation of key genes involved in gibberellin biosynthesis and promotes higher gibberellin levels

The significant boost in plant height, early flowering, leaf width and surface area could be associated to the stimulation of cellular division and expansion triggered by gibberellins (Ogawa *et al.*, 2003; de Lucas *et al.*, 2008). GAs are hormones normally involved in development, including plant height (Ross *et al.*, 1993). The first step in GA biosynthesis is the cyclization of GGPP in the plastid carried out by copalyl diphosphate synthase (CPS) to produce copalyl diphosphate; subsequenty ent-kauren synthase (KS) catalyzes the synthesis of ent-kauren (Sun and Kamiya, 1994). GA12-aldehyde is then formed by the action of cytochrome-P450-dependent mono-oxygenases. GA12-aldehyde is the first GA produced, and is the precursor of all other GA types. The GA20-oxidases and GA3-oxidases participate in the formation of the bioactive forms, GA1 and/or GA4 (Hedden and Kamiya, 1997; Hedden and Phillips, 2000). Overexpression of GA20-oxidases in Arabidopsis and tobacco induce longer hypocotyls and stem internodes as well as increments in GA4 levels (Coles *et al.*, 1999). Transgenic tobacco plants overexpressing GA20ox genes, flower early and have higher biomass accumulation, lignin formation and photosynthesis rate (Biemelt *et al.*, 2004). Through the expression of the carotenogenic gene *Dclcyb1*, we obtained similar results in the phenotypic parameters as well as the increment in active GA4 content and in the transcript abundance of *Ntga20ox1*. The enhancement in the production of active GA4 contradicted previous observations reporting that the overexpression of carotenogenic genes impairs GA synthesis in plants. In tomato, the expression of the fruit paralog *psy*, produced dwarf plants that was associated with a reduction of GA and a slight increase in ABA, as a response to the competition for GGPP (Fray *et al.*, 1995). In tobacco, a strong rise in *psy* expression led to a dwarf phenotype and a decrease in total pigment content (Busch *et al.*, 2002). Recently, Tata *et al.* (2015) produced *Haggpps* transgenic tobacco plants with higher biomass and early flowering as a consequence of the increment in GA, but without affecting chlorophyll and with a significant reduction in carotenoids levels. On the contrary, we induced an increment in active GA4 and in the phenotypical parameters associated with this hormone such as plant height, leaf size and whole plant biomass and early flowering (Griffiths *et al.*, 2006; Gupta and Chakrabarty, 2013) by the expression of *Dclcyb1* in tobacco (Table 3), accompanied by an increase in chlorophyll and carotenoid levels. The fact that by manipulating carotenoid synthesis we produced a positive effect on gibberellin levels indicates that there is crosstalk between these pathways in tobacco. This is supported by the significant induction of *Ntcps, Ntks* and *Ntga20ox1* transcripts, key genes in GA synthesis, which is most likely the reason for the increment of GA4. Even though in Arabidopsis the overexpression of *Atcps* and *Atks* by themselves does not increase the content of bioactive GAs (Fleet *et al.*, 2003), in our tobacco lines this increment is accompanied by changes in other genes in the GA pathway, like *Ntga20ox1*, which may explain the different results in the GA content. Additionally, the differences between tobacco and Arabidopsis may reflect the differences in metabolic regulations from one species to another. Furthermore, GA20-oxidases (but not GA3-oxidases) are known to be limiting enzymes in many species, including tobacco (Gallego-Giraldo *et al.*, 2008), as was confirmed partially in our transgenic *Dclcyb1* plants. The quantification of bioactive gibberellins showed differences in the content of GA4 but not GA1 between transgenic and control plants (Fig. 5). This is consistent with an induction of *GA20ox* genes and an increase of GA4 and not GA1 by overexpressing a GA20-oxidase in tobacco plants (Vidal *et al.*, 2001), presumably by competition with GA13ox for the substrate GA_12_. In those transgenic plants, the level of GA4 (the active GA produced from the non-13-hydroxylation pathway) in the shoot of transgenic plants was 3–4 times higher than in control plants, whereas that of GA1, formed via the early-13-hydroxylation pathway (the main GA biosynthesis pathway in tobacco), was not incremented (Vidal *et al.*, 2001). These differences were enough to induce a substantial increase in plant height and leaf length (up to twofold). Therefore, we can conclude that *Dclcyb1* produces an up-regulation of key genes involved in gibberellin biosynthesis and promotes higher gibberellin levels. In addition, the evidence that GA4 is indeed involved in the enhanced growth and better fitness of *Dclcyb1* transgenic lines is further supported by the GA inhibition assay in tobacco seedlings (Supplementary Fig. S4).

### Feedback regulation mechanisms of carotenoid biosynthesis

The modulation of endogenous carotenogenic gene transcripts after the expression or down-regulation of one gene of the pathway seems to be a common feedback mechanism. Retrograde signaling is required to regulate the expression of these carotenogenic genes that in turn leads to a coordination in the metabolic flux of the pathway. *Psy* is one of carotenogenic genes that is mostly regulated at the transcriptional level by this feedback mechanism (Ruiz-Sola and Rodríguez-Concepción, 2012, Arango *et al.*, 2014).

Consequently, this permits us to propose that the boost in the carotenoid, chlorophyll and gibberellin pathways, as a consequence of *Dclcyb1* expression, is the result of a positive feedback loop, mediated by a molecule derived from β-carotene that is produced in plastids and that acts through the retrograde signaling to induce the transcription of the nuclear encoded carotenogenic and isoprenoid biosynthetic genes. Carotenoids by themselves are very unlikely to function as immediate signaling metabolites in the regulation of gene expression because of their lipophilicity. Enzymatic cleavage of β-carotene by NCED and carotenoid cleavage deoxygenases (CCD) produces apocarotenoids in the cytoplasm such as ABA and strigolactones (SLs), respectively. Exogenous ABA treatment up-regulates *psy* expression in Arabidopsis roots (Ruiz-Sola *et al.*, 2014) and so this hormone could be a retrograde signaling molecule. However, neither ABA levels nor *Ntnced3* expression are induced significantly in *Dclcyb1* transgenic tobacco lines (Fig. 4, Supplementary Fig. S5), suggesting that in this case another signal molecule could be involved. SLs, mycorradicin and bixin, among other apocarotenoids, are generated by the action of CCDs on β-carotene, zeaxanthin and lycopene, respectively. Recently Lätari et al. (2015) determined that the unchanged carotenoid levels in leaves of Arabidopsis *Atpsy* overexpresser lines is a result of the production of higher levels of C_13_ apocarotenoid glycosides (AGs) produced by CCD4 through the cleavage of specific xanthophyll molecules. CCD4 was also reported to be involved in the accumulation of specific linear carotenes in zds mutants, which act as signals to regulate the expression of chloroplast- and nucleus-encoded genes during development (Avendano-Vazquez *et al.*, 2014). Other candidates derived from the cleavage of β-carotene are several short chain molecules produced by chemical oxidation of β-carotene by singlet oxygen (_1_O^2^) that are collectively designated as carotenoid reactive electrophile species (RES) [e.g., β-cyclocitral (β-CC) and dihydroactinidiolide (dhA)] (Tian, 2015). Both, β-CC and dhA are volatile compounds that elicit gene expression in the nucleus via a retrograde signaling mechanism to trigger the acclimation of Arabidopsis plants to high light (Ramel *et al.*, 2012; Shumbe *et al.*, 2014). The small metabolite 2-C-methyl-D-erythritol 2, 4-cyclodiphosphate (MEcPP), an isoprenoid intermediate of the MEP pathway, is a dynamic abiotic stress inducible plastid-to-nucleus communication signal that also induces the expression of selected unfolded protein response genes facilitating plastid-ER communication (Walley *et al.*, 2015). Interestingly, genes from the MEP pathway, *Ntdxs2* and *Ntggpps,* are also upregulated in our transgenic plants (Fig. 4). DXS, the first enzyme in the MEP pathway is essential for the production of isoprenoid precursors and for induction of carotenoid synthesis (Estevez *et al.*, 2001). Crosstalk between the isoprenoid precursor and the carotenoid pathway has been determined before (Rodríguez-Concepción, 2006; Cazzonelli and Pogson, 2010). Specifically, the up-regulation of *psy* in de-etiolated Arabidopsis seedlings increases carotenoid levels and produces a feedback mechanism that results in the accumulation of the DXS protein (Rodríguez-Villalon *et al.*, 2009). On the contrary, an unexpected reduction in total carotenoids and a lower PSY protein level was obtained in roots of carrots that express the AtCYP97genes, which is proposed as a negative feedback inhibition produced by carotenoids downstream of α-carotene (Arango *et al.*, 2014). The mechanism underlying the phenomenon by which the overexpression of *Dclcyb1* is capable of inducing the expression of other genes remains to be determined. Nevertheless, considering that the mechanism of regulation of the carotenoid flux may vary from one species to another and that the results obtained may also depend on the nature of the gene and its transcript abundance, it is conceivable that a β-carotene-derived molecule could act as a retrograde signal to induce feedback-activating isoprenoid precursors, which is sufficient to explain the increment in carotene, gibberellins and chlorophyll amounts in *DcLcyb1*-overexpressing tobaccos. 

**Fig. 6.**
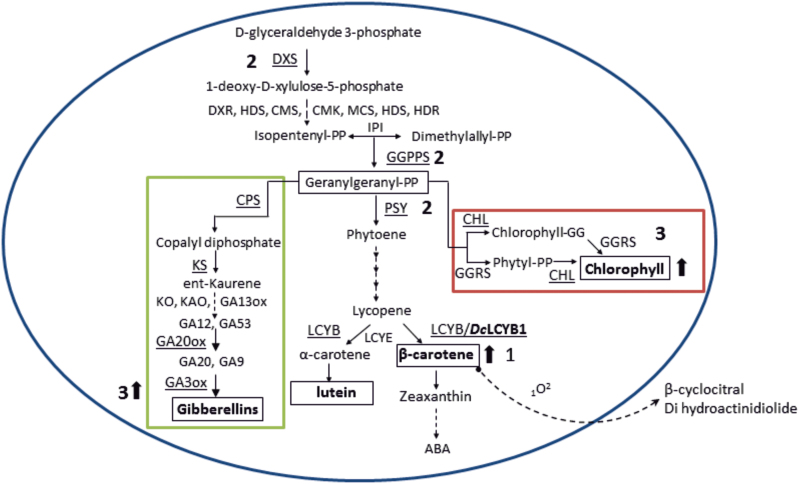
Scheme of a proposed model for boosting carotenoid, gibberellin and chlorophyll synthesis in *Dclcyb1* transgenic tobacco. Carotenoid, chlorophyll and gibberellin biosynthesis pathways are presented. The over expression of *Dclcyb1* causes a greater production of β-carotene (1) that may be cleaved by singlet oxygen to produce apocarotenoids such as β-cyclocitral or dihydroactinidiolide that may act as retrograde signaling molecule(s) to activate the expression of endogenous *psy* genes (2), or of *dxs* and *ggpps* genes involved in early isoprenoid production (2), raising probably the synthesis of the common precursor, GGPP, resulting in higher accumulation of gibberellins, chlorophylls and carotenoids (3). (This figure is available in colour at *JXB* online.)

Taking the results presented here and elsewhere together, we propose that *Dclcyb1* results in higher β-carotene levels (and lutein; step 1, Fig. 6) which in turn permits the production of signaling molecules, such as β-cyclocitral or dihydroactinidiolide that modulates the transcription of endogenous *Ntpsy1* and *Ntpsy2* (step 2, Fig. 6) or may up-regulate directly the expression of *Ntdxs2* or *Ntggpps* from the isoprenoid pathway (step 2, Fig. 6), producing an increase in the common precursor GGPP (step 2, Fig. 6) and triggering the rise in total carotenoids, chlorophyll and gibberellin levels (step 3, Fig. 6). Unlike other reports in tomato and Arabidopsis (Fray *et al.*, 1995; Qin *et al.*, 2007; Tata *et al.*, 2015), tobacco plants may have sufficient precursor molecules that are available for the three common pathways, boosting fitness in these transgenic plants.

Finally, the work presented here provides an alternative approach for genetic manipulation of agronomically valuable crops to increase plant fitness by means of the overexpression of a single carrot carotenogenic gene.

## Supplementary data

Supplementary data are available at *JXB* online.

Fig. S1. β-carotene and chlorophyll content in transgenic T0 tobacco lines.

Fig. S2. Expression of endogenous carotenogenic genes in leaves of Dclcyb1 transgenic T0 tobacco plants.

Fig. S3. Expression of genes involved in the biosynthesis of gibberellins in leaves of Dclcyb1 transgenic T1 tobacco plants.

Fig. S4. Gibberellin synthesis inhibitor assay.

Fig. S5. Abscisic acid levels in transgenic tobacco plants.

Table S1. Gene-specific primer sequences used in this work.

Table S2. Comparison of fitness in T0 Dclcyb1 transgenic lines.

Table S3. Comparison of fitness in T2 Dclcyb1 transgenic lines.

Table S4. Biomass measurements in T2 Dclcyb1 transgenic lines

Supplementary Data
