# Supplementary material for: Increased Nicotiana tabacum fitness through positive regulation of carotenoid, gibberellin and chlorophyll pathways promoted by Daucus carota lycopene β-cyclase (Dclcyb1) expression
Source: J Exp Bot. 2016 Feb 18;67(8):2325–38. doi: 10.1093/jxb/erw037 (PMC4809289; doi:10.1093/jxb/erw037)
Supplement: Supplementary Data [file supp_67_8_2325__index.html]

Increased Nicotiana tabacum fitness through positive regulation of carotenoid, gibberellin and chlorophyll pathways promoted by Daucus carota lycopene β-cyclase (Dclcyb1) expression — Increased Nicotiana tabacum fitness through positive regulation of carotenoid, gibberellin and chlorophyll pathways promoted by Daucus carota lycopene β-cyclase (Dclcyb1) expression — Supplementary Data 

# Increased *Nicotiana tabacum* fitness through positive regulation of carotenoid, gibberellin and chlorophyll pathways promoted by *Daucus carota* lycopene β-cyclase (*Dclcyb1*) expression

## Supplementary Data

Data files

- supplementary\_figures\_S1\_S5\_tables\_S1\_S4.pdf - Supplementary Data
