# Supplementary material for: Increased Nicotiana tabacum fitness through positive regulation of carotenoid, gibberellin and chlorophyll pathways promoted by Daucus carota lycopene β-cyclase (Dclcyb1) expression
Source: J Exp Bot. 2016 Feb 18;67(8):2325–38. doi: 10.1093/jxb/erw037 (PMC4809289; doi:10.1093/jxb/erw037)
Supplement: Supplementary Data [file supp_erw037_supplementary_figures_S1_S5_tables_S1_S4.pdf]

Supplementary figures

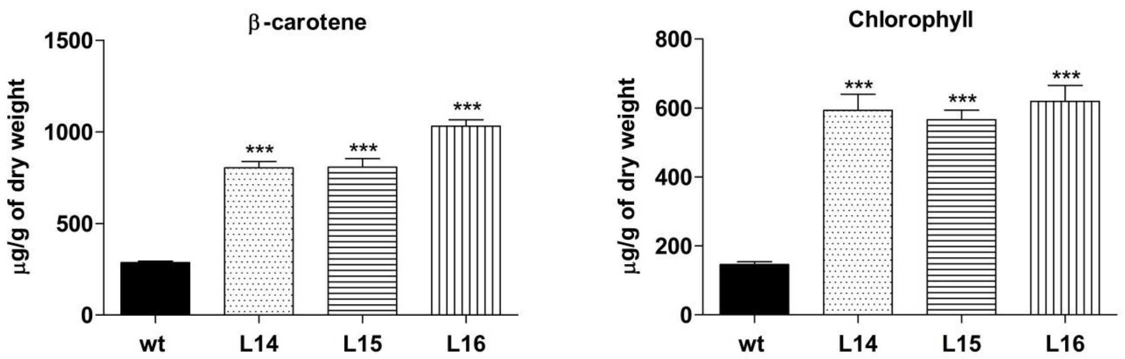

Fig S1 β-carotene and chlorophyll content in transgenic T0 tobacco lines. Amounts of β-carotene (left) and chlorophyll (right) in leaves of three month-old wild type (wt) and *Dclcyb1* transgenic (L14, L15 and L16) T_0 lines were determined by HPLC. Columns and bars represent the means and SD (n = 3). Asterisks indicate significant differences between transgenic lines and the wild type plant using non-paired two tailed Student t-tests, *** p<0.001.

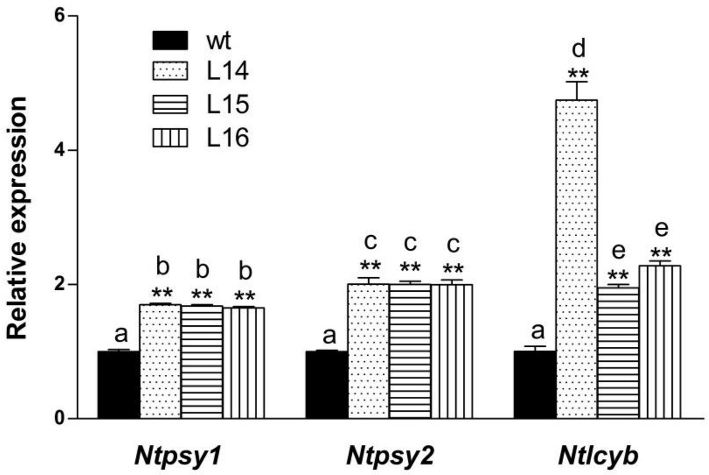

Fig S2 Expression of endogenous carotenogenic genes in leaves of *Dclcyb1* transgenic T_0 tobacco plants. Expression of *Ntpsy1, Ntpsy2* and *Ntlcyb* was normalized to *Actin* in qRT-PCR measurements. Wild type (wt) plant expression was selected as calibrator. Columns and bars represent the means and SD (n = 3). Asterisks and letters indicate significant differences between transgenic lines and the wild type plant using non-paired one and two tailed Student t-tests, **: $p<0.01$.

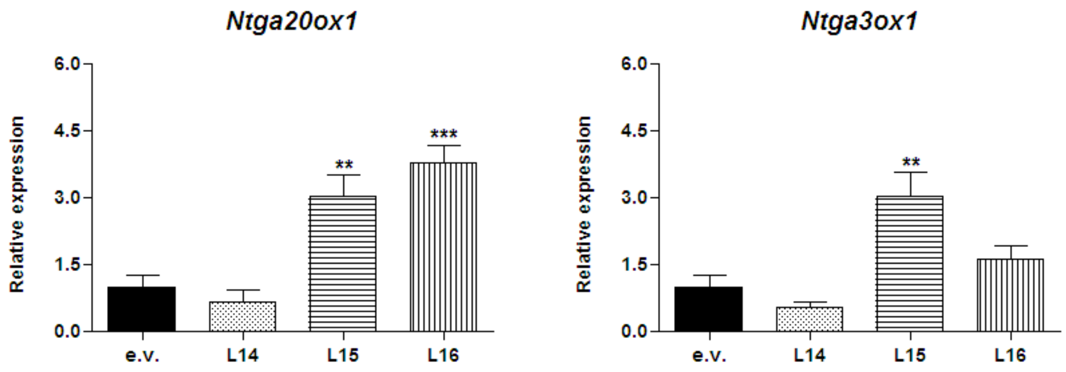

Fig S3 Expression of genes involved in the biosynthesis of gibberellins in leaves of *Dclcyb1* transgenic T1 tobacco plants. Expression of *NtGA20ox1* (AB012856.1) and *NtGA3ox1* (AB032198) was normalized to *Actin* in qRT-PCR measurements. The expression of plants transformed with the empty vector (e.v.) was used as calibrator. Columns and bars represent the means and SD (n = 3). Asterisks indicate significant differences between transgenic lines and the e.v. plants using non-paired one and two tailed Student t-tests, **: p<0.01.

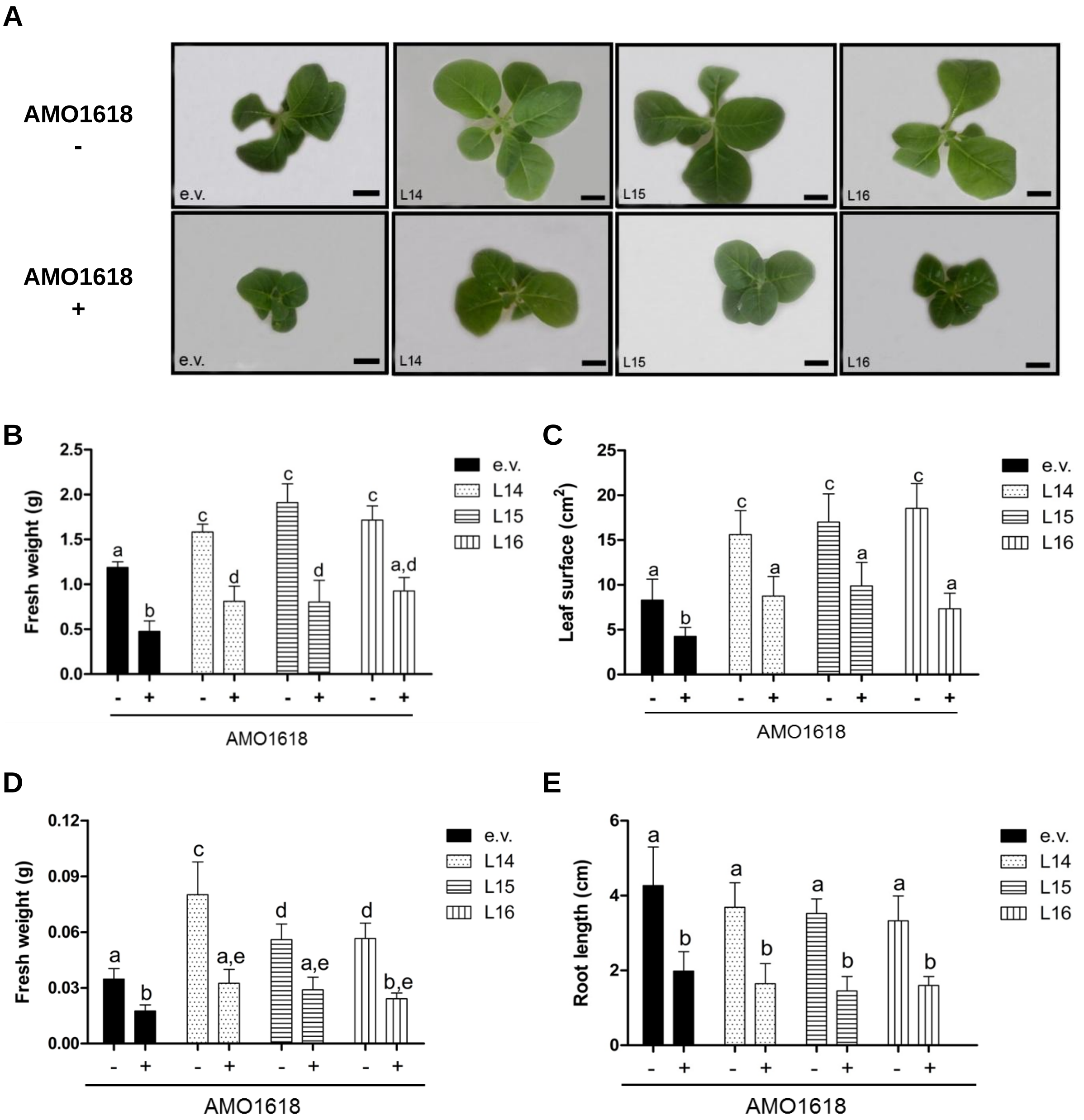

Fig S4 Gibberellin synthesis inhibitor assay. A) One-month old empty vector (e.v.) and *Dclcyb1* (L14, L15 and L16) transgenic T_1 tobacco lines were grown in the absence (-; upper) or presence (+; bottom) of the gibberellin synthesis inhibitor AMO1618 (100 µM) and photographed after one month of culture in magenta boxes. B) Fresh weight and C) leaf surface area for e.v., L14, L15 and L16 transgenic lines growing with (+) or without (-) AMO1618 during one month. Tobacco seedlings (T_1) transformed with the empty vector (e.v.) or *Dclcyb1* (L14, L15 and L16) were grown in vertical plates in the presence (+) or absence (-) of the gibberellin synthesis inhibitor AMO1618 (100 µM) during 21 days. (D) Fresh weight. (E) Root length. Columns and bars represent the means and SE (n = 16). Letters indicate significant differences between *DcLcyb1* transgenic and e.v. lines, as determined by ANOVA analysis with Tukey´s post-test, p<0.0001. Scale bar represents 2 cm.

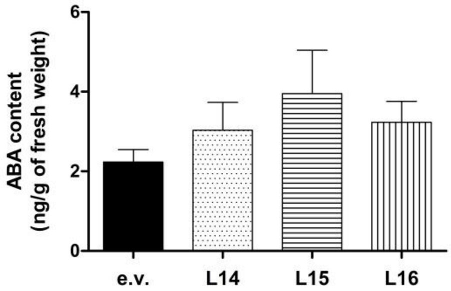

Fig S5 Abscisic acid levels in transgenic tobacco plants. ABA content in leaves of 1 month-old empty vector (e.v.) and *Dclcyb1* (L14, L15 and L16) transgenic T_1 tobacco plants. Three biological replicates and two technical replicates were analyzed. Non-paired one and two tailed Student t-tests were performed.

Table S1. Gene-specific primer sequences used in this work.

Name	NCBI Access	Sequence (5'→3')	Reference
***DcLcyb*F**	DQ192190.1	TTGACCTTCCTTTGTATGACCCGTC	*Moreno et al.*, 2013.
***DcLcyb*R**		TCCTGCCTCAGAAACTTGTTGTGC	
***DcLb1* F**	DQ192190.1	TTGACCTTCCTTTGTATGACCCGTC	This work
***DcLb1* R**		TCCTGCCTCAGAAACTTGTTGTGC	
***Nt*Actin F**	EU938079.1	GTATGTCGCCATTCAAGCCGTTCT	This work
***Nt*Actin R**		ACGGAGGATAGCATGTGGCAAAGCAT	
***NtUbiq* F**	U66264.1	GCTCGAGGACGGCAGAAC	This work
***NtUbiq* R**		CTTGGGCTTGGTGTAGGTCTTC	
***Nt18S* F**	AJ236016.1	CTGAGAAACGGCTACCACATCCA	This work
***Nt18S* R**		CACCAGACTTGCCCTCCAATGG	
***NtPsy1* F**	JF461341.1	GGAACCAAGCTAATGACCCCAGAGAGA	This work
***NtPsy1* R**		GGCCGCCCACTGAAAATATCTTCC	
***NtPsy2* F**	JX101475.1	TCAGAGATGTTGGAGAAGATGC	This work
***NtPsy2* R**		GCTTCAATCTCGTCCAATATCTTG	
***NtLcyb* F**	KC484706.1	CCGTGTTAAATTCCACCACGCCAA	This work
***NtLcyb* R**		GAAGCCAGTTGCATCAAGCACCAC	
***NtGgpps* F**	GQ911583.1	GTATTGGGTTGTTGTTTCAAGTTGTGGAG	This work. Consensus for all gene members
***NtGgpps* R**		GCAATCAATGGAGCTGCTTTGTCTGGATC	
***NtDxs1* F**	EU650419.1	GCCTTAGATGGACTTCTTGATGGCAAGT	This work
***NtDxs1* R**		TGTTAAACACTGTTGCTGCAATGTGAGAT	
***NtDxs2* F**	JQ085430.1	AGAGCATAACAAAGCAAATTGGACCTC	This work
***NtDxs2* R**		CTCCTCAAAAAGAGTTGAACAAGAAGCAC	
***NtChl* F**	FJ905101.1	ATCAAATATGGGTGCTTCTTCTTGGAGG	This work
***NtChl* R**		ATTATGTCAGGTGTAAGGGTGCCGAACA	
***NtCps* F**	AB170034.1	ACACTAAAGCTGACATGGATACCAAAGG	This work
***NtCps* R**		CATAAGTGCAAAGGCAGTAGAAGATGGA	
***NtKs* F**	JQ413246.1	TACTTACTACCGTGGTTGATGACTTCTTG	This work
***NtKs* R**		CTTCATCTCCAATCTCACAAATAGTGCTT	
NtGA20ox1 F	AB012856.1	TGTAGCACGAGAACTTCC	Gallego-Giraldo, et al., 2008
NtGA20ox1 R		ACGGCATGCTTCACCAACA	

NtGA3ox1F	AB032198	CGGCTTTGTCCCCTCTA	Gallego-Giraldo, et al., 2008
NtGA3ox1R		CTTATCGAGTTTAGCCAACTTGCA	
***NtNced* F**	JX101472.1	CTTTACCAAAAACAGCCGACCCACG	This work
***NtNced* R**		CACCAATGGCTTTAGGAAAAACAGGACG	

Table S2. Comparison of fitness in T_0 *Dclcyb1* transgenic lines.

Lines	Plant height (cm)	Stem diameter (cm)	Leaf length (cm)	Leaf wide (cm)	Leaf surface (cm^2)	Nodal interspace (cm)	Leaf number
wt	30.8	0.8	12.8[a]	8.1[a]	103.7[a]	**1.8[a]**	20
L14	73.8	0.92	**22.0[b]**	**12.3[b]**	**270.6[b]**	**4.2[b]**	14
L15	75.2	0.9	**25[b]**	**13.2[b]**	**330[b]**	**3.9[b]**	16
L16	70.7	0.89	**25[b]**	**13[b]**	**325[b]**	**3.9[b]**	12

*a, b: Non-paired two tailed Student t-tests ($p<0.05$) were performed for all the transgenic lines (wt, L14, L15 and L16) and the differences are represented by letters and significant values are boldfaced. Three representative leaves were used. The mean data are presented (n=3), except for plant height, stem diameter and leaf number parameters where n=1.

Table S3. Comparison of fitness in T_2 *Dclcyb1* transgenic lines.

Lines	Plant height (cm)	Stem diameter (cm)	Leaf length (cm)	Leaf wide (cm)	Leaf surface (cm^2)	Nodal interspace (cm)	Leaf number
e.v.	31.8^a	0.8^a	12.8^a	8.1^a	104.8^a	1.8^a	21^a
L14	**57.8^b**	0.75^a	**22.0^b**	**11.8^b**	**215.3^b**	**3.1^b**	**15^b**
L15	**58.2^b**	0.79^a	**21.9^b**	**13.1^b**	**213.8^b**	**3.1^b**	**15^b**
L16	**55.7^b**	0.79^a	**20.2^b**	**12.9^b**	**207.0^b**	**3.0^b**	**15^b**

*a, b: Non-paired two tailed Student t-tests ($p<0.05$) were performed for all the transgenic lines (e.v., L14, L15 and L16) and the differences are represented by letters and significant values are boldfaced. Three representative leaves from 7 different plants were used. The mean data are presented (n=7).

Table S4. Biomass measurements in T_2 *Dclcyb1* transgenic lines.

Lines	Biomass FW/leaf (g)	Biomass FW/stem (g)	Biomass FW/plant (g)	Biomass DW/leaf (g)	Biomass DW/stem (g)	Biomass DW/plant (g)	Seed production/plant (g)
e.v.	16.3[a]	10.1	26.4[a]	1.8[a]	1.6[a]	3.4[a]	0.7[a]
L14	**23.0[b]**	**17.5[b]**	**40.5[b]**	2.3[a]	**2.4[b]**	**4.7[b]**	**1.5[b]**
L15	**21.6[b]**	**17.9[b]**	**39.9[b]**	2.0[a]	**2.2[b]**	4.2[a]	**1.6[b]**
L16	**22.6[b]**	**18.7[b]**	**41.3[b]**	2.1[a]	**2.2[b]**	4.3[a]	**1.5[b]**

*a, b: Non-paired two tailed Student t-tests ($p<0.05$) were performed for all the transgenic lines (e.v., L14, L15 and L16) and the differences are represented by letters and significant values are boldfaced. The mean data are presented (n=7).
